# Supplementary material for: Mechanisms governing the pioneering and redistribution capabilities of the non-classical pioneer PU.1
Source: Nat Commun. 2020 Jan 21;11:402. doi: 10.1038/s41467-019-13960-2 (PMC6972792; doi:10.1038/s41467-019-13960-2)
Supplement: Supplementary file 7 — Source data [file 41467_2019_13960_MOESM7_ESM.zip › Source_Data/FigureS2/FigureS2B_MotifScanOutput/KmeansCluster8/homerResults/motif1.similar.html]

motif1

## Information for motif1

G
A
T
C
C
T
G
A
A
T
G
C
G
C
A
T
G
A
C
T
A
G
T
C
A
G
T
C
A
G
C
T
A
G
T
C
G
C
A
T
G
C
A
T
G
A
C
T
  
Reverse Opposite:  

C
T
G
A
C
G
T
A
C
G
T
A
T
C
A
G
T
C
G
A
C
T
A
G
T
C
A
G
C
T
G
A
C
G
T
A
T
A
C
G
G
A
C
T
C
T
A
G
  

|  |  |
| --- | --- |
| p-value: | 1e-869 |
| log p-value: | -2.002e+03 |
| Information Content per bp: | 1.593 |
| Number of Target Sequences with motif | 1195.0 |
| Percentage of Target Sequences with motif | 79.03% |
| Number of Background Sequences with motif | 4070.4 |
| Percentage of Background Sequences with motif | 8.97% |
| Average Position of motif in Targets | 100.7 +/- 39.4bp |
| Average Position of motif in Background | 101.5 +/- 76.9bp |
| Strand Bias (log2 ratio + to - strand density) | -0.0 |
| Multiplicity (# of sites on avg that occur together) | 1.40 |
| Motif File: | file (matrix) reverse opposite |

### Similar de novo motifs found

|  |  |  |  |  |  |  |  |
| --- | --- | --- | --- | --- | --- | --- | --- |
| Rank | Match Score | Redundant Motif | P-value | log P-value | % of Targets | % of Background | Motif file |
| 1 | 0.992 | A G T C C T G A A T G C C G A T A G C T A G T C A G T C G A C T A G T C G A C T G A C T G C A T G A T C | 1e-849 | -1956.014145 | 83.27% | 11.52% | motif file (matrix) |
| 2 | 0.974 | C T G A A T G C C G A T A C G T A G T C A G T C A G C T A G T C G C A T G A C T G A C T | 1e-823 | -1895.948070 | 73.48% | 7.51% | motif file (matrix) |
| 3 | 0.968 | C T G A C G T A T C A G T C G A C T A G A C T G C G T A C G T A T A C G G A C T | 1e-782 | -1802.614362 | 76.19% | 9.50% | motif file (matrix) |
| 4 | 0.975 | A G T C G A T C C T G A A T G C C G A T A C G T A G T C A G T C G A C T A G T C G C A T G A C T G A C T G A C T | 1e-741 | -1707.297669 | 72.75% | 8.86% | motif file (matrix) |
| 5 | 0.962 | C G T A T C A G T C A G C T A G A C T G C G T A G C T A T A C G G A C T | 1e-649 | -1496.231724 | 74.40% | 11.95% | motif file (matrix) |
| 6 | 0.917 | T C A G T C G A A C T G A C T G C G T A C G T A T A C G A G C T | 1e-637 | -1467.235965 | 77.98% | 14.39% | motif file (matrix) |
| 7 | 0.949 | A T G C A C G T A C G T A G T C A G T C A G C T A G T C G A C T G C A T G C A T G A T C | 1e-562 | -1295.232928 | 90.87% | 27.85% | motif file (matrix) |
| 8 | 0.898 | C T G A A C T G A C T G C G T A C G T A A T C G A C G T | 1e-525 | -1209.407106 | 72.49% | 14.85% | motif file (matrix) |
| 9 | 0.946 | A T C G C G A T C G A T A G T C A G T C G A C T A G T C G C A T G A C T G C A T A G T C A G C T | 1e-515 | -1187.987287 | 94.25% | 34.43% | motif file (matrix) |
| 10 | 0.955 | A T G C C G A T C A G T T A G C A G T C A G C T A G T C G C A T G C A T G C A T | 1e-511 | -1177.336042 | 94.97% | 35.70% | motif file (matrix) |
| 11 | 0.903 | C T A G T C G A T C A G T A C G G C T A G C T A T A C G C G A T C T A G | 1e-506 | -1166.480514 | 75.79% | 17.72% | motif file (matrix) |
| 12 | 0.910 | C A G T G T C A C G T A T A C G C T G A A C T G A C T G G T C A C T G A T C A G G A T C | 1e-502 | -1156.713284 | 79.37% | 20.50% | motif file (matrix) |
| 13 | 0.894 | C T A G C T G A C T A G A C T G C G T A G C T A C T A G A G C T T C G A T A C G | 1e-486 | -1119.261400 | 71.69% | 15.83% | motif file (matrix) |
| 14 | 0.885 | C T G A G C T A C G T A T A C G T G C A T C A G A C T G C T G A T C G A | 1e-484 | -1116.396549 | 67.59% | 13.40% | motif file (matrix) |
| 15 | 0.891 | A T G C A C G T A C G T A G T C A G T C A G C T A G T C | 1e-453 | -1045.300398 | 80.36% | 23.62% | motif file (matrix) |
| 16 | 0.895 | C T G A A T C G A C G T A C G T A G T C A G T C A G C T A C G T G T A C | 1e-448 | -1033.844499 | 90.54% | 33.95% | motif file (matrix) |
| 17 | 0.936 | G A T C C G T A A T G C C G A T A C G T A T G C G A T C A G C T A G T C G C T A C G T A T C G A T C G A G T C A | 1e-440 | -1015.049446 | 55.22% | 8.53% | motif file (matrix) |
| 18 | 0.870 | C T G A C T A G A C T G C G T A C G T A T C A G C G A T C T A G T C A G C G A T C G A T | 1e-409 | -942.515733 | 58.93% | 11.32% | motif file (matrix) |
| 19 | 0.850 | C T G A C A T G T A C G T C G A C G T A T C A G C G A T C T A G T A C G G C A T C G A T C G A T | 1e-368 | -848.340523 | 50.00% | 8.32% | motif file (matrix) |
| 20 | 0.885 | C G T A T C A G T C G A G T C A G T C A A T C G C T G A C T A G A C T G T C G A C G T A T A C G G C A T | 1e-364 | -838.949391 | 46.69% | 7.00% | motif file (matrix) |
| 21 | 0.867 | G C T A G C A T A G T C G T A C G C T A A T G C G C A T G A C T A T G C A G T C G A C T G A T C G A C T | 1e-360 | -829.773815 | 55.89% | 11.50% | motif file (matrix) |
| 22 | 0.889 | G A C T G C A T A T G C A G T C A G C T A T G C G A C T C G A T | 1e-359 | -828.682080 | 82.67% | 31.25% | motif file (matrix) |
| 23 | 0.773 | A G T C C G T A A G T C C G A T A C G T A G T C A G T C | 1e-251 | -578.647996 | 53.51% | 15.05% | motif file (matrix) |
| 24 | 0.794 | T C G A G C T A T C G A C T G A G T C A C T A G C T G A C T A G C A T G C T G A G C T A T C G A T A C G | 1e-224 | -517.491001 | 43.65% | 10.60% | motif file (matrix) |
| 25 | 0.751 | C G T A T C A G C G A T G C A T G A T C G A T C G C A T A T G C A C G T A G C T A G C T A G C T | 1e-133 | -307.611417 | 16.14% | 1.84% | motif file (matrix) |
| 26 | 0.787 | A C G T A T C G A G T C A C G T A C G T A G T C A G T C A C G T A G T C | 1e-131 | -302.245944 | 30.03% | 7.92% | motif file (matrix) |
| 27 | 0.835 | A C T G T C G A C G T A A C T G A C T G A T C G A C T G T G C A C T G A T C A G A C G T C G T A C T A G | 1e-122 | -281.448878 | 21.89% | 4.36% | motif file (matrix) |
| 28 | 0.844 | C T A G T C G A C T A G C T A G C G T A C G T A T A G C G A C T | 1e-106 | -245.670908 | 24.87% | 6.52% | motif file (matrix) |
| 29 | 0.752 | T C G A C G T A C G T A C T A G A T G C C G T A A C T G C T G A C G T A T A C G G C A T C T A G | 1e-100 | -232.475718 | 17.06% | 3.11% | motif file (matrix) |
| 30 | 0.753 | G A T C C G T A A T G C G C A T A C G T A G T C G A C T T A C G G A T C G C A T G A C T G A C T A G C T A G C T | 1e-98 | -227.725905 | 16.60% | 3.00% | motif file (matrix) |
| 31 | 0.753 | G C T A G C T A T C A G C T G A T C A G C A T G T G C A C T G A C A T G A T C G T G C A | 1e-95 | -220.440848 | 24.93% | 7.18% | motif file (matrix) |
| 32 | 0.756 | C T A G C T G A C G T A C T G A C A T G A C T G T C A G A C T G C T G A T C G A T A C G C G A T A C T G | 1e-90 | -209.142779 | 7.87% | 0.45% | motif file (matrix) |
| 33 | 0.799 | C A T G C T A G C A T G A T C G C G T A A C T G A C T G C G T A C T G A T C A G T A G C G C A T T C A G | 1e-90 | -208.480489 | 21.56% | 5.68% | motif file (matrix) |
| 34 | 0.808 | A T G C G A C T C A G T A G T C A T G C G A C T T G A C T A C G A C G T A C G T | 1e-76 | -177.277325 | 14.02% | 2.75% | motif file (matrix) |
